# On Climate and Sea Voyages in the Treatment of Tuberculosis

**Published:** 1899-06-10

**Authors:** 


### ON CLIMATE AND SEA VOYAGES IN THE TREATMENT OF TUBERCULOSIS.

Speaking at the same Congress, Sir Hermann Weber explained the share which climate can take in the treatment of tuberculosis. After describing the peculiarities of mountain climates, inland climates of low altitude, climates of the coast, and sea-voyages, he discussed the general question of the indications for the use of the different climates in different cases of tuberculosis. The difficulty of determining on the proper climate for individual cases depends largely on the fact that while tuberculosis occurs in various forms and with complications of considerable variety, the constitutions of the individuals affected are also very different. In all medical work we have to consider the constitution as well as the disease, and in dealing with tuberculosis this is especially the case. Much must depend on whether the patient has a weak or a strong constitution, and in deciding this point the family history is of even more importance than it is in regard to any presumed hereditary tendency to tuberculosis. If the patient belongs to a long-lived family we may presume that he possesses a strong constitution, even although other members of the family have died of tuberculosis; whereas if he belongs to a short-lived family we generally have to deal with an originally weak constitution. Again, in regard to the same question, personal history is of importance. Those who from youth up always feel better, are more energetic, can eat better, and put on weight in warm weather, but in cold weather become less capable of work, generally belong to the class of weak constitutions, as also do those who from childhood onwards have always had high fever with every slight illness, have recovered with difficulty, and who always have moist hands, whereas those who have always done better in the cold possess stronger constitutions. Now, when it becomes a matter of choice of climate, we must be guarded in recommending high altitudes and long sea voyages to patients with weak constitutions, but must give the preference rather to warm, sunny, and sheltered places. On the other hand, in strong, somewhat torpid constitutions mountain climates are to be preferred, and occasionally long sea voyages, and much can also be done by keeping them continuously in the open air, and by good feeding and regulated exercise. The choice of a climate is, of course, largely influenced by the amount, the character, and the locality of the disease, but the constitutional state must always be considered. Finally, it must be borne in mind that, although the cure of tuberculosis during the early stages is possible in all healthy climates, some climates have advantages for various cases over others; for instance, notably those of high altitudes. But climate by itself without careful medical supervision is generally insufficient, and the patient's blind reliance upon climate often leads to errors, to aggravation of the disease, and to death. For the majority of patients, then, treatment in sanatoria should be preferred, while for the treatment of the poor it is a necessity.

